# Posterior parietal cortex evaluates visuoproprioceptive congruence based on brief visual information

**DOI:** 10.1038/s41598-017-16848-7

**Published:** 2017-11-30

**Authors:** Jakub Limanowski, Felix Blankenburg

**Affiliations:** 1 Neurocomputation and Neuroimaging Unit, Department of Education and Psychology, Freie Universität Berlin, Berlin, Germany; 2 Center for Cognitive Neuroscience Berlin, Freie Universität Berlin, Berlin, Germany

## Abstract

To represent one’s upper limbs for action, the brain relies on a combined position estimate based on visual and proprioceptive information. Monkey neurophysiology and human brain imaging suggest that the underlying operations are implemented in a network of fronto-parietal and occipitotemporal cortical areas. Recently, a potential hierarchical arrangement of these areas has been proposed, emphasizing the posterior parietal cortex (PPC) in early multisensory comparison and integration. Here, we used functional magnetic resonance imaging (fMRI) and a virtual reality-based setup to briefly (0.5 s) present healthy human participants photorealistic virtual hands, of matching or nonmatching anatomical side, or objects at the same or a different location than their real hidden left or right hand. The inferior parietal lobe (IPL) of the left PPC showed a significant preference for congruent visuoproprioceptive hand position information. Moreover, the left body part-selective extrastriate body area (EBA; functionally localized) significantly increased its coupling with the left IPL during visuoproprioceptive congruence vs. incongruence. Our results suggest that the PPC implements early visuoproprioceptive comparison and integration processes, likely relying on information exchange with the EBA.

## Introduction

Controlling the body’s actions in a constantly changing environment is one of the brain’s most important tasks. During manual actions, the brain integrates sensory information from at least the visual and proprioceptive modalities to estimate the current state of the hand^1–3^. However, visual and proprioceptive information is not always integrated - experiments manipulating the degree of visuoproprioceptive congruence have demonstrated that the seen limb position must be anatomically plausible and sufficiently similar to the felt (proprioceptive) limb position to affect the multisensory body representation^4–10^. Correspondingly, behavioral experiments have shown that visual body part stimuli that are congruent with the current body position more rapidly enter visual awareness^11,12^. In sum, these findings suggest that visuoproprioceptive congruence is evaluated in the light of a pre-existing body representation^13–15^.

As a likely candidate brain region for such comparison and evaluation processes, neurophysiological recordings in monkeys^6,16,17^ have identified the posterior parietal cortex (PPC). Recently, the human PPC and also the body-selective extrastriate body area (EBA^18^) have been analogously implied in comparing seen and felt hand positions^8,19–23^. It has been speculated that these regions may form a potential pathway for rapid (recurrent) processing of visual hand information for action^11,12,24^. Rapid and early processing and comparison of visuoproprioceptive hand information involving information exchange between PPC and EBA would also correspond to theoretical models of the “rubber hand illusion” (RHI^4^), which assume an early evaluation of body-related multisensory input in PPC before it is further processed in premotor areas^13–15^.

**Figure 1:**
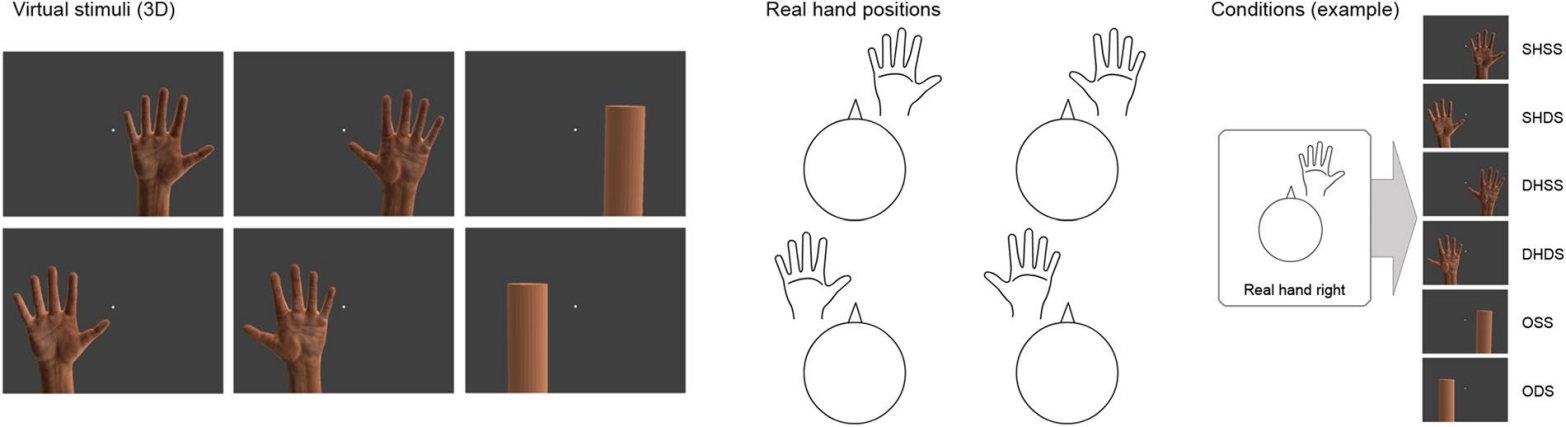
Experimental stimuli and conditions. In a virtual reality-based setup, participants were repeatedly presented a three-dimensional photorealistic left or right virtual hand (in a palm facing position), or a virtual cylindrical object, for 0.5 s either left or right of a central fixation dot (separated by a 2.5–6.5 s jittered interstimulus interval). In each run, participants adopted one of four possible real hand positions, i.e., placed their left or right hand in the left or right hemifield above their face, with the palm facing them (schematically shown). The real hands were thus plausibly aligned with the seen stimuli, and always hidden from view by the stereoscopic goggles used for visual presentation. Depending on the current real hands’ position, the seen stimulus could be assigned to one of the following six conditions (example for the real hand on the right-position shown): same hand at same side (SHSS, i.e., visuoproprioceptive congruence), same hand at different side (SHDS), different hand at same side (DHSS), different hand at different side (DHDS), object at same side (OSS), or object at different side (ODS).

To investigate such rapid and early visuoproprioceptive comparisons, we used functional magnetic resonance imaging (fMRI) and a virtual reality-based setup to test whether very brief presentations (0.5 s) of virtual hands that could match the real hands’ anatomical laterality and position would specifically engage the PPC and EBA. In each of four experimental runs, the participant adopted a different hand position: either the left or the right hand was placed on top of the scanner head coil above the left or right half of their face. Via stereoscopic goggles, participants were presented a photorealistic left or right virtual hand (in a palm facing position), or a virtual cylindrical object in 3D, which roughly matched the virtual hand in terms of volume, position, and color; the real hand was always hidden from view by the goggles. All visual stimuli were presented left or right from a central fixation dot. Thus, depending on the current real hands’ position, the visual stimuli could be a same (matching) hand, a different hand, or an object, each presented on the same or different side as the currently raised real hand (Fig. 1). Visuoproprioceptive congruence was thereby only given when the laterality *and* the location of the virtual hand matched those of the real hand. We hypothesized that this condition would relatively more strongly engage the PPC and EBA.

## Results

Throughout the experiment, participants had to count randomly presented 300 ms long pulsations of the fixation dot, and report their count after each run, which was intended to ensure constant central fixation. Participants on average detected 96.6% (SD = 4.6%) of these pulsations with a false alarm rate of 2.1% (SD = 2.5%), which suggests that they maintained fixation throughout the experiment. Correspondingly, contrasting all stimulus presentations in the left vs. right visual hemifield (and vice versa) revealed significant (*p* < 0.05, corrected for multiple comparisons) activation differences exclusively in the respective contralateral primary and associative visual cortex.

### Body part-selective brain areas

**Figure 2:**
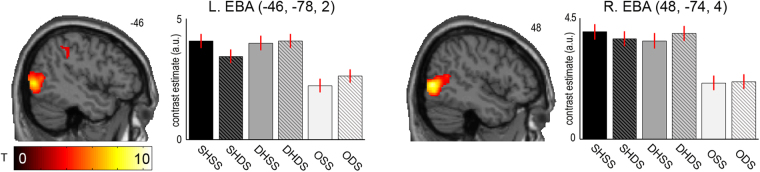
EBA localizer. The SPM shows significant (*p* < 0.05, corrected for multiple comparisons) activation differences to visual presentation of hands vs. objects in the left and right middle occipital gyrus, matching previously reported locations of the EBA. The bar plots show the contrast estimates with associated standard errors for each condition at the respective peak voxel. SHSS = same hand seen at same side (as the currently raised real hand), SHDS = same hand at different side, DHSS = different hand at same side, DHDS = different hand at different side, OSS = object at same side, ODS = object at different side. The corresponding SPM is available at https://neurovault.org/images/56830/.

As an implicit functional localizer of the body part-selective EBA, we contrasted the presentation of hands vs. objects, which as expected revealed significant (*p* < 0.05, corrected for multiple comparisons) activations at locations in the bilateral middle occipital gyri previously reported for the EBA^18,25^, spanning to the primary and secondary visual cortex, as well as in the bilateral PPC and left dorsal premotor cortex. See Fig. 2.

### Brain areas sensitive to visuoproprioceptive congruence

**Figure 3:**
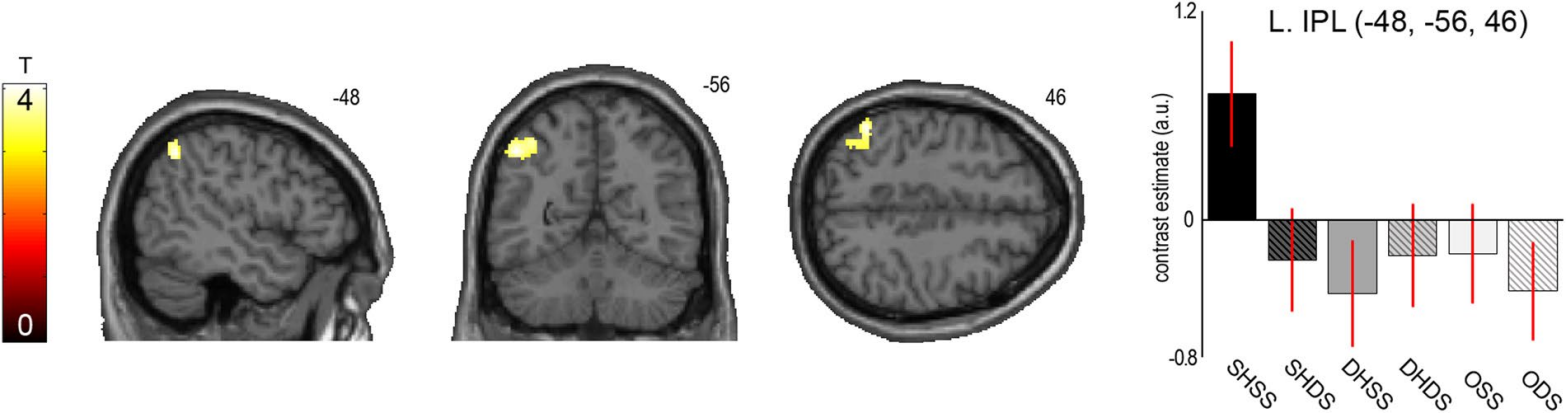
Visuoproprioceptive congruence effects. The SPM shows significant (*p* < 0.05, corrected for multiple comparisons) activation differences obtained from contrasting visuoproprioceptively congruent presentations (SHSS) versus all other virtual hand presentations (SHDS, DHSS, DHDS) in the left IPL. The bar plots show the contrast estimates with associated standard errors for each condition at the cluster’s peak voxel (also showing responses to object presentations for comparison). SHSS = same hand seen at same side (as the currently raised real hand), SHDS = same hand at different side, DHSS = different hand at same side, DHDS = different hand at different side, OSS = object at same side, ODS = object at different side. The corresponding SPM is available at https://neurovault.org/images/56826/.

In our main analysis we looked for brain regions that would prefer visuoproprioceptive congruence, i.e., the presentation of a matching virtual hand at the matching location with respect to the real raised hand (SHSS condition) versus all other virtual hand presentations (SHDS, DHSS, DHDS). The corresponding contrast revealed a significant cluster of activation in the left inferior parietal lobule (IPL; *x* = −48, *y* = −56, *z* = 46, *T* = 4.05, *p* < 0.05, corrected for multiple comparisons), spanning to the intraparietal sulcus (IPS), see Fig. 3. The same region of the left IPL was also significantly sensitive to the presentation of matching hands vs. objects (SHSS vs. objects contrast, *p* < 0.05, corrected for multiple comparisons). To test whether the visuoproprioceptive selectivity in the left IPL generalized across real hand sides, we calculated the respective SHSS vs. other hands contrasts for the real right hand and real left hand separately; a global conjunction of these contrasts indeed revealed a corresponding significant activation at the same IPL location (*x* = −40, *y* = −66, *z* = 44, *T* = 2.14, *p* < 0.05, corrected for multiple comparisons).

No other activations survived correction for multiple comparisons, but there were uncorrected (*p* < 0.001) activations in the right IPL (*x* = 44, *y* = −64, *z* = 36, *T* = 4.17) and at the junction of the left precentral sulcus and inferior frontal gyrus, potentially including the left ventral premotor cortex (PMv; *x* = −42, *y* = 20, *z* = 36, *T* = 3.68).

When contrasting all presentations of the same vs. different virtual hand laterality, i.e., (SHSS + SHDS) > (DHSS + DHDS), we observed significant activations in more posterior parts of the bilateral IPL (see Supplementary Fig. S1). The other comparisons (other hands vs. RHI; different vs. same virtual hand laterality presented; main effect of stimuli presented in the same vs. different hemifield as the currently raised real hand) yielded no significant activations.

### Connectivity analysis (psychophysiological interactions)

**Figure 4:**
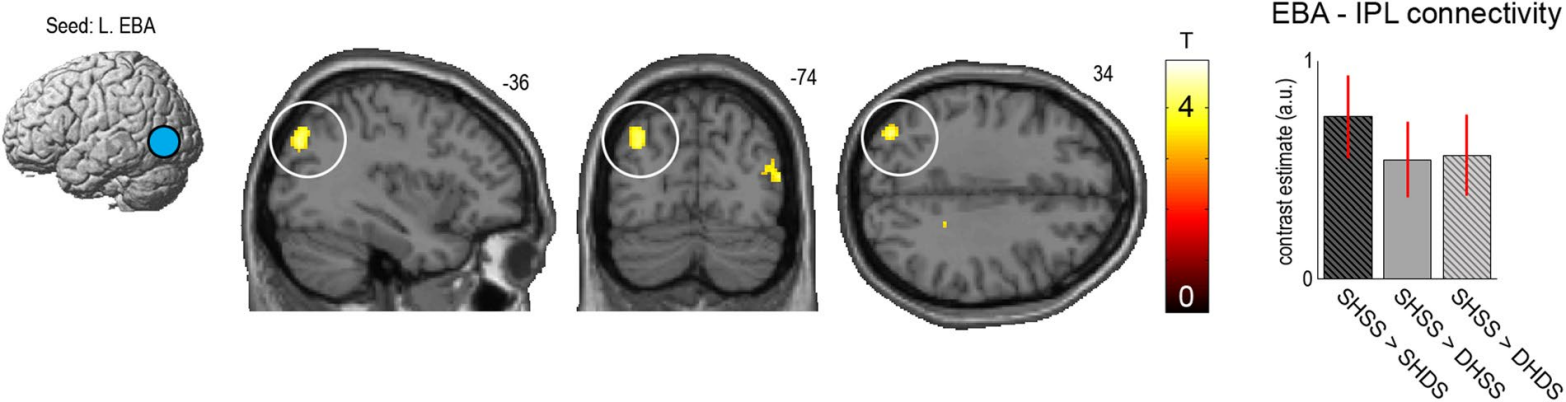
Results of the connectivity analysis. Activity in the left body part-selective EBA (seed region defined based on contrast hands vs. objects presentation, schematically depicted here, see Fig. 2) was significantly more strongly coupled with activity in the left IPL during visuoproprioceptive congruence (*p* < 0.05, corrected for multiple comparisons, marked by white circle; for display purposes the SPM is thresholded at *p* < 0.001, uncorrected), i.e., there was relatively increased EBA-IPL connectivity during presentations of a matching virtual hand at the same side as the real hand was located (SHSS) versus all other virtual hand presentations (SHDS, DHSS, and DHDS). The bar plot shows the relative increase in functional connectivity between the left EBA (seed region) and the left IPL during SHSS vs. each of the other hand presentations (contrast estimates at the IPL’s peak voxel for each PPI in arbitrary units, with standard errors). The corresponding SPM is available at https://neurovault.org/images/56828/.

Based on our hypothesis that visuoproprioceptive comparisons would involve information exchange between left-lateralized body part-selective visual regions (i.e., the EBA) and the PPC^13,19–23^, we next examined relative changes in the left EBA’s connectivity (seed region defined based on the orthogonal body parts vs. objects contrast, see Fig. 2) under presentation of a visuoproprioceptively congruent virtual hand (SHSS) vs. the other virtual hand presentations (SHDS, DHSS, DHDS) using psychophysiological interaction analysis. This analysis indeed revealed an overall significantly increased coupling of the left EBA to the same regions of the left IPL as identified in the main analysis (*x* = −34, *y* = −76, *z* = 36, *T* = 4.37, *p* < 0.05, corrected for multiple comparisons, see Fig. 4). No other activations were statistically significant. A global conjunction contrast confirmed that the increase in coupling of the left EBA to the left IPL was significantly consistent across each of the individual comparisons (*p* < 0.05, corrected for multiple comparisons, see Supplementary Fig. S2). An analogous analysis with the seed region placed in the right EBA did not reveal any significant coupling changes.

## Discussion

We found that the left IPL showed significantly higher activation levels to very brief visual presentations of a virtual hand that matched the current configuration of the participant’s hidden real hand (i.e., a virtual hand of matching laterality and at the same location), compared to the presentation of nonmatching hands, matching hands at different locations, and non-hand objects. Moreover, the left body-part selective EBA significantly increased its coupling with the same regions of the left IPL during visuoproprioceptively congruent vs. incongruent virtual hand presentations. Our results suggest that posterior parietal and body-part selective occipitotemporal regions work together to rapidly evaluate visuoproprioceptive congruence—a crucial first step for multisensory estimation of limb position for body representation and action control.

The increased activation of the IPL by conditions with visuoproprioceptive congruence fits with related fMRI studies on the RHI, which likewise reported increased activation of this region by synchronous vs. asynchronous tactile stimulation of a congruently vs. incongruently positioned fake hand^8,20,25^. Recently, we were able to isolate visuoproprioceptive comparisons from the visuotactile stimulation used to induce the RHI, and showed that the left IPL preferentially responds to visual presentations of a right hand in a corresponding vs. rotated position at the same spatial location^22^. Here, we extend these findings by showing that even very brief visual presentations trigger the multisensory mechanisms in the IPL. Further, our results suggest that this visuoproprioceptive selectivity in the IPL also takes into account spatial location congruence, and generalizes to both real hand sides.

We did not find significant PMv activation differences, which are typically observed during the RHI^8,20,25,26^. This can be well explained by the fact that we focused on very brief visual stimulus presentations in the absence of visuotactile stimulation. The PMv contains neurons with visual and tactile receptive fields centered on specific body parts, especially the upper limbs^5,27,28^. The PMv significantly increases its activity after full induction of the RHI by several seconds of synchronous visuotactile stimulation, and its activity usually correlates with the intensity of the illusion^8,20^. Correspondingly, a hierarchical processing of multisensory body-related information has been suggested, with the PMv at its potentially highest level implementing the representation of the fake hand and the space around it for action, based in turn on the earlier multisensory comparisons in the PPC^13,20,22,25^. Here, we demonstrated that visuoproprioceptive comparisons in the absence of touch, triggered by only 0.5 s visual input, indeed significantly engage the PPC. Tentatively, our results thus support the proposed cortical hierarchy of body representation, with early essential multisensory comparisons and integrations in the PPC. Our research could be extended with more temporally fine-grained methods like electro- or magnetoencephalography to investigate potentially resulting (top-down) changes in the processing of proprioceptive information, and by additional manipulations of proprioceptive information, which here was held constant throughout scanning. Further, in posterior parts of the PPC, we observed a general preference for presentations of a visual hand of same vs. different laterality (also at a nonmatching spatial location), which could indicate a certain tolerance for visuoproprioceptive location incongruence. Future work could directly investigate this by parametrically manipulating visuoproprioceptive congruence.

Our second main finding was the relatively increased functional connectivity between the same areas of the IPL and the left EBA (which we identified as body part-selective in our sample with our implicit functional localizer) during visuoproprioceptive congruence, which can be interpreted as indicating a relative increase in communication between these areas^29^. Previous imaging studies had shown relatively increased connectivity of the PPC and EBA during the RHI^20,25^, but as noted these studies could not isolate the early visuoproprioceptive comparisons from other illusion-related effects such as the comparisons of seen and felt touches (the manipulation used to induce the RHI). Here, we demonstrate a relatively increased coupling between the PPC and EBA during very brief presentation of proprioceptive-congruent vs. incongruent visual hand stimuli.

It has previously been speculated that the PPC and EBA may form a potential rapid pathway for action-relevant processing of visual hand information^24^, and that recurrent processing from PPC to EBA may increase visual awareness of the hand image^11^. However, since its discovery as a visually body part-selective region^18^, the EBA has also been shown to be involved in the preparation and execution of movements^19,21,23^, which may suggest multisensory (visual and proprioceptive) input to the EBA. An important question for future work is whether visuoproprioceptive comparisons first take place in the PPC, based on visual information provided by the EBA—or whether (and to which extent) they already occur in the EBA itself.

To conclude, we showed that the IPL of the PPC rapidly evaluates visuoproprioceptive congruence of hand position information, and likely does so by relatively increasing communication with the EBA. Our results add strong support to proposals that these two brain regions form an important, and hierarchically early circuit for body representation.

## Methods

### Participants

21 healthy volunteers (13 male, mean age = 24 years, range = 19–37, with normal or corrected-to-normal vision) participated in the experiment after giving written informed consent. The experiment was approved by the ethics committee of the Freie Universität Berlin, and conducted in accordance with this approval and the relevant guidelines and regulations.

### Experimental design and procedure

During the experiment, participants lay inside the fMRI scanner with one of their hands (left or right) placed in a palm facing position on a foam pad on top of the MR-head coil; the participant’s arm was supported by foam pads until reaching a relaxed position that did not require the participant to actively hold the arm elevated. In each of the four experimental runs, the participant adopted a different hand position: either the right or the left hand was placed right or left from fixation (i.e., atop the left or right half of their face), respectively. Velcro markers on the foam pad helped the participant to reach the desired hand positions. The order of hand positions was counterbalanced across participants.

We used stereoscopic goggles (VisuaSTIM, 800 × 600 pixels, 30° eye field) and the Blender graphics software package (http://www.blender.org) to present participants a three-dimensional photorealistic left or right virtual hand (in a palm facing position), or a virtual cylindrical object, which roughly matched the virtual hand in terms of volume, position, and color; the real hand was always hidden from view by the goggles. See Fig. 1.

We used an event-related design, in which the virtual hand or object was presented for 0.5 s either left or right of a central fixation dot. Each of the 6 stimuli (right hand in right hemifield, right hand in left hemifield, left hand in right hemifield, left hand in left hemifield, object in right hemifield, and object in right hemifield) was presented 10 times per run in randomized order. The presentations were separated by a jittered interstimulus interval (2.5–6.5 s) and additional null events (10 per run), resulting in about 6 minutes run length.

To promote constant fixation, we included a catch trial detection task. Throughout each run, the fixation dot pulsated briefly (30% increase in size for 300 ms), unpredictably 6 to 16 times per run. Participants had to report the number of counted pulsations verbally to the experimenter after each run.

### FMRI data acquisition, preprocessing, and analysis

The fMRI data were recorded using a 3 T scanner (Tim Trio, Siemens, Germany), equipped with a 12-channel head coil. T2*-weighted images were acquired using a gradient echo-planar imaging sequence (3 × 3 × 3 mm³ voxels, 20% gap, matrix size = 64 × 64, TR = 2000 ms, TE = 30 ms, flip angle = 70°). For each participant, we recorded 4 runs à 180 functional image volumes and a T1-weighted structural image (3D MPRAGE, voxel size = 1 × 1 × 1 mm³, FOV = 256 × 256 mm², 176 slices, TR = 1900 ms, TE = 2.52 ms, flip angle = 9°). FMRI data were preprocessed and analyzed using SPM12 (www.fil.ion.ucl.ac.uk/spm/). Artifacts at the slice-level were corrected using the ArtRepair toolbox^30^. Images were corrected for slice acquisition time differences, realigned and resliced, normalized to MNI space and resliced to 2 mm voxel size, spatially smoothed with an 8 mm full width at half maximum Gaussian kernel, detrended (this step was skipped for the images used for the PPI analysis, see below)^31^, and images featuring excessive movement were interpolated (ArtRepair toolbox). We fitted a general linear model (GLM, 64 s high-pass filter) to each participant with convolved regressors modeling the aforementioned conditions and catch trials, as well as the realignment parameters as regressors of no interest.

Depending on the real hands’ position, the seen stimulus could be assigned to one of the following six conditions: same hand at same side (SHSS), same hand at different side (SHDS), different hand at same side (DHSS), different hand at different side (DHDS), object at same side (OSS), or object at different side (ODS). For each of these conditions, we calculated the corresponding first-level contrast images, which were entered into a second-level 3 × 2 factorial design with the factors *Stimulus congruence (Same hand, Different hand, Object)* and *Spatial congruence (Same side, Different side)*. We used one-sample t-tests to evaluate additional first-level contrast images testing for the retinotopy of visual presentation and the effect of catch trials.

Based on previously reported increased connectivity of the left PPC and the left body-selective EBA^20,22,25^, we analyzed the connectivity (i.e., changes in the statistical dependencies of BOLD signal time series) between the left body-part selective EBA (seed region) and voxels in the whole brain under presentation of a matching virtual hand at the same side as the real hand was located (SHSS) versus all other virtual hand presentations (SHDS, DHSS, DHDS)) by means of psychophysiological interaction (PPI) analysis^29^. PPIs for each comparison (SHSS > SHDS, SHSS > DHSS, and SHSS > DHDS) were all calculated as follows. For each participant, the four experimental runs were concatenated into a single data sequence. The seed region was limited to all significant voxels of the EBA that were within 4 mm radius of the group-level maximum obtained from the respective contrast body parts vs. objects in the main GLM analysis (see Fig. 2). The seed region’s BOLD time series was then extracted as the first eigenvariate of those voxels. We then calculated the interaction between the psychological context (i.e., weighting the SHSS conditions with +1 and the SHDS, DHSS, or DHDS conditions with −1) and the extracted seed region BOLD signal time series to reveal voxels across the whole brain, in which activity would be more strongly correlated with the EBA’s activity under visuoproprioceptively congruent (SHSS) versus the respective incongruent virtual hand presentation. The first-level PPI GLMs included the extracted seed region time course, the psychological context variable, their interaction (the PPI), as well as all regressors modelling the other conditions to account for their specific effects. These resulting first-level contrast images of the three individual PPIs were jointly evaluated on the group level using a main effect and global conjunction contrast in a one-way within-subject ANOVA. For completeness, although prior work suggests a left-lateralization of the PPC-EBA circuit^13,19–23^, we also conducted an analogous analysis for a seed region set in the right EBA.

The obtained activations were assessed for statistical significance applying a threshold of *p* < 0.05, family-wise error (FWE) corrected for multiple comparisons on the cluster level with a cluster-defining threshold of *p* < 0.001. Based on prior hypotheses about communication between body-selective EBA and parietal areas^11,20,21,25^, we assessed the statistical significance of activations in the left IPL obtained from the connectivity analysis from the EBA seed region (see above) applying peak-level FWE correction for multiple comparisons within a restricted IPL search region of interest (ROI) defined via all IPL voxels showing an effect of SHSS vs. other hand presentations in the main analysis at *p* < 0.005, uncorrected. We only display activations that survived correction for multiple comparisons; the corresponding statistical parametric maps (SPMs) are projected onto SPM’s template brain with a cluster extent threshold of 10 voxels. Reported coordinates are in MNI space; the SPM Anatomy toolbox^32^ was used for anatomical reference where possible.

### Data availability

The authors declare that the data supporting the findings of this study are available within the paper. The SPMs of all reported contrasts (and their unthresholded versions) are available at https://neurovault.org/collections/3153/. Individual fMRI datasets are available from the corresponding author upon request.

## Electronic supplementary material

Supplementary material
