# Supplementary material for: Posterior parietal cortex evaluates visuoproprioceptive congruence based on brief visual information
Source: Sci Rep. 2017 Nov 30;7:16659. doi: 10.1038/s41598-017-16848-7 (PMC5709509; doi:10.1038/s41598-017-16848-7)
Supplement: Supplementary file 1 — Supplementary material [file 41598_2017_16848_MOESM1_ESM.pdf]

Supplementary Material

Posterior parietal cortex evaluates visuoproprioceptive congruence based on brief visual information

Jakub Limanowski and Felix Blankenburg

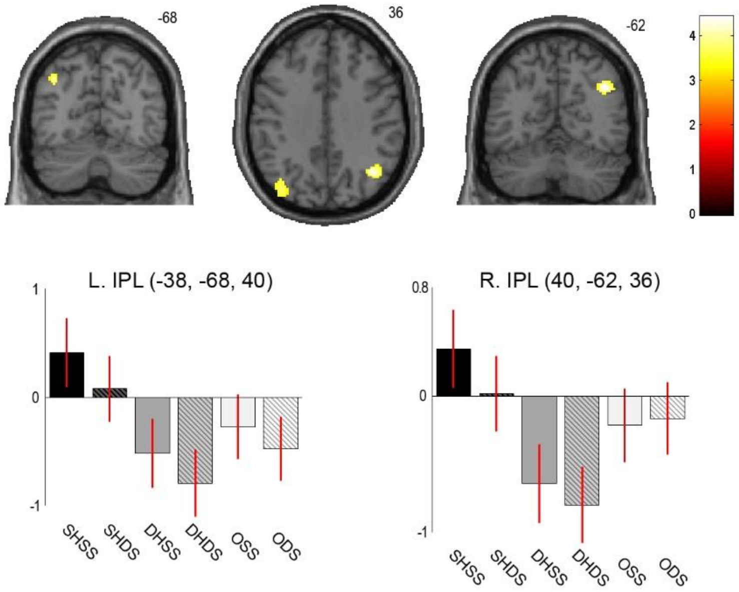

Figure S1. A contrast of visual presentations of the same vs. different virtual hand laterality, i.e., (SHSS + SHDS) > (DHSS + DHDS) revealed significant ($p < 0.05$, corrected for multiple comparisons) activations in the left and right IPL. The bar plots show the contrast estimates with associated standard errors for each condition, including responses to object presentation, at the respective peak voxel. SHSS = same hand seen at same side (as the currently raised real hand), SHDS = same hand at different side, DHSS = different hand at same side, DHDS = different hand at different side, OSS = object at same side, ODS = object at different side.

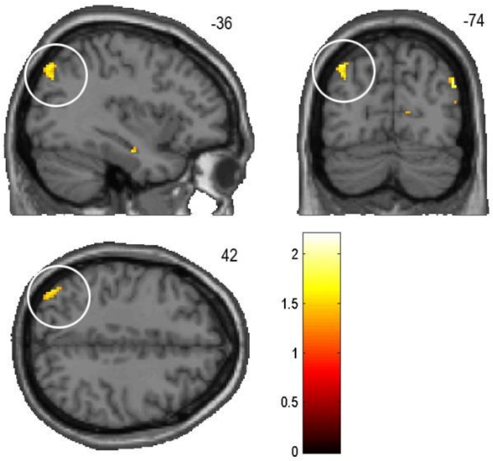

Figure S2. A global conjunction of the three individual PPI contrasts, testing for consistency of the main effect of increased EBA-IPL functional connectivity reported in Figure 4, revealed significant ($p < 0.05$, corrected for multiple comparisons) activations at the same IPL locations ($x = -36$, $y = -74$, $z = 46$, $T = 1.78$, $Z = 3.83$).
